# Insect biorefinery: a green approach for conversion of crop residues into biodiesel and protein

**DOI:** 10.1186/s13068-017-0986-7

**Published:** 2017-12-14

**Authors:** Hui Wang, Kashif ur Rehman, Xiu Liu, Qinqin Yang, Longyu Zheng, Wu Li, Minmin Cai, Qing Li, Jibin Zhang, Ziniu Yu

**Affiliations:** 1 State Key Laboratory of Agricultural Microbiology, National Engineering Research Center of Microbial Pesticides, College of Life Science and Technology, Huazhong Agricultural University, Wuhan, People’s Republic of China; 2 College of Science, Huazhong Agricultural University, Wuhan, People’s Republic of China; 3 College of Animal Science and Technology & College of Veterinary Medicine, Huazhong Agricultural University, Wuhan, People’s Republic of China; 4 Livestock and Dairy Development Department, Punjab, Pakistan

**Keywords:** Yellow meal worm, Black soldier fly, Corn stover, Biorefinery, Biofuel, Biodiesel

## Abstract

**Background:**

As a major lignocellulosic biomass, which represented more than half of the world’s agricultural phytomass, crop residues have been considered as feedstock for biofuel production. However, large-scale application of this conventional biofuel process has been facing obstacles from cost efficiency, pretreatment procedure, and secondary pollution. To meet the growing demands for food, feed, and energy as the global population continues to grow, certain kinds of insects, many of which are voracious feeders of organic wastes that may help address environmental, economic, and health issues, have been highlighted as a source of protein and fat.

**Results:**

The biorefinery studied includes initial corn stover degradation by yellow mealworm (*Tenebrio molitor* L.), followed by a second stage that employs black soldier fly (*Hermetia illucens* L.), to utilize the residues produced during the first stage. These two insect-based biorefinery yielded 8.50 g of insect biomass with a waste dry mass reduction rate of 51.32%, which resulted in 1.95 g crude grease from larval biomass that produced 1.76 g biodiesel, 6.55 g protein, and 111.59 g biofertilizer. The conversion rate of free fatty acids of crude grease into biodiesel reached 90%. The components of cellulose, hemicellulose, and lignin contained in corn stover hydrolyzed harmoniously, resulting in declines of 45.69, 51.85, and 58.35%, respectively. Moreover, fluctuations in lipid, protein, and reducing sugar were also analyzed.

**Conclusion:**

The investigation findings demonstrated that successive co-conversion of corn stover by insects possessing different feeding habits could be an attractive option for efficient utilization of lignocellulosic resources, and represents a potentially valuable solution to crop residues management, rise of global liquid energy, and animal feed demand. 
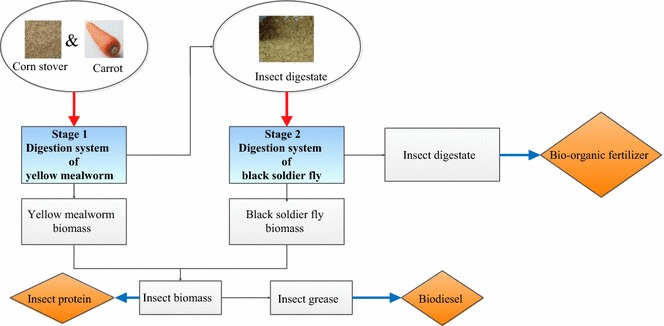

## Background

The rapid expansion of the world economy, an increase of the global population, and improvement of the standard of living have created an enormous burden on conventional energy resources and the environment [1]. The worldwide usage of oil and other liquid fuels was predicted to grow from 90 million barrels per day (b/d) to 100 million b/d in the 8 years from 2012 to 2020, and to 121 million b/d by 2040 [2, 3]. Global energy consumption, which was 549 quadrillions British thermal unit (Btu) in 2012, is likely to rise to 815 quadrillions Btu by 2040, an increase of 48% [2, 3]. In the contexts of depleting fossil reserves, higher oil prices, energy security, and environmental impacts (such as CO_2_ emission and global warming), the modern societies require the development of sustainable, economic, and energy-efficient processes [4]. These concerns have led to a global policy shift toward the use of biomass as renewable and low-cost raw materials for liquid energy generation [5].

Crop residues left on the field represent more than half of the world’s agricultural phytomass [6]. Globally, the contributions from agricultural residues were estimated to be 27.2, 21.9, and 26.7% for corn, wheat, and rice straw, respectively [7]. Corn stover (CS) was the most abundant of all such crop residues [8], and over the last 10 years, considerable research has been conducted to improve its utilization [9, 10]. CS has been identified as a suitable raw material for biofuel production due to its high cellulose content and its vast availability [11, 12].

Crop residues, such as CS, wheat, and rice straw are mainly composed of lignocellulose, which could be used for biofuel production [13]. The first generation of biofuels from food crops has drawbacks e.g. greenhouse gas emissions and increasing food costs. Production of second-generation biofuels from lignocellulosic biomass, mainly bioethanol, remains challenging in view of special equipment requirements that accompany it, higher costs, heavy water consumption, and complex production technology [14]. Also, industrial production of ethanol requires titers greater than 40 g/L for high-efficiency refining [15]. To some extent, cellulosic ethanol production is currently not economically feasible, and it is environmentally unfriendly. Thus, there is an urgent need to identify other potential sources and technologies for biofuel production and lignocellulose utilization.

A more economically feasible and environmentally friendly approach is the use of insect to convert crop residues into biomass and simple organic materials: a concept called biotransformation [15, 16]. Fortuitously, insects belonging to the order Coleoptera—such as the yellow meal worm (YMW) and *Tenebrio molitor* L., and Diptera—such as the black soldier fly (BSF) and *Hermetia illucens* L. can efficiently degrade organic matters, transforming wastes into larval biomass [17–19]. The YMW larvae were reported to contain 23–47% fat [20, 21], and are an important scavenger of decayed milled cereals and grains under humid and poor conditions. They eat storage products and have been distributed all over the world [20, 22]. Production of environmentally friendly biodiesel from YMW fed with decayed vegetable matter was first reported by Zheng et al. [23]. Similarly, BSF larvae (BSFL) contain 20–40% fat [24, 25]. Biodiesel production using BSFL fed with animal manure [26], rice straw [27], restaurant wastes [28], and corncob [29] have been reported. Insect fat has been proposed as a promising resource for biodiesel production [30]. Therefore, interest in insects as lipid feedstock producer has increased, particularly due to its capacity to grow on diverse sources of biomass, such as low-quality waste material from animal and plant origins [29, 30]. They can accumulate a large amount of saturated fatty acids (i.e., C18 and C16) with desirable physical and chemical properties, such, as kinematic viscosity, calorific value, oxidation stability, which are conducive to further conversion into biodiesel [31]. The resulting defatted larval biomass can also be used as a protein source for poultry, aquaculture, and livestock.

The conversion of biomass into marketable products, fuels, and chemicals via biorefining is similar to traditional refineries [32]. The main difference is in the raw materials, petroleum refining uses crude oil, and biorefineries use biomass. Biorefineries are being established in wide-ranging industrial scales and focusing on processing multiple products. These designs typically employ a significant product yield and profit from a single source of raw materials, but the scarcity of materials and environmental impacts must be considered. The integrated biorefinery configuration can be utilized for the production of high value-added products [33]. In this study, we highlight the multi-insect biorefinery for production of biofuel and protein, transforming crop residues into insect biomass. The biorefinery studied includes initial corn stover degradation by yellow mealworm (*Tenebrio molitor* L.), followed by a second stage that employs black soldier fly (*Hermetia illucens* L.), to utilize the residues produced during the first stage. A multi-insect (YMW and BSF) biorefinery system for operating CS was established and optimized. Our objective was to make neonate insect production technology to be economic and environmentally friendly while producing high-quality biofuel, protein, and biofertilizer.

## Methods

### Source of insects and waste biomass

YMW larvae used in the present investigation were obtained from the colony maintained in the insect rearing base of the National Engineering Research Center of Microbial Pesticides, Huazhong Agricultural University (HZAU), Wuhan, China. Fly larvae were collected from the colony of BSF (*H. illucens*, L. Wuhan strain) which was established in 2008 and maintained at the State Key Laboratory of Agricultural Microbiology of HZAU, Wuhan, China. This Wuhan strain has been sustained in a greenhouse for 8 years before it was used in this study [34].

Corn stover used in this study was collected from the maize breeding farm of HZAU. Before being used, CS was dried at 60 °C for 6 days in an electric oven (DHG-06-200B, Haisheng Equipment Co., Ltd,China). Then, it was chopped and ground using a laboratory blender (BJ-1000A, Baijie Electric Appliance Co., Ltd, China), sieved for size (< 3 mm), and stored in a drying oven at 60 °C. Dumped defective carrot waste for moisture supplement was obtained from the local vegetable market of HZAU, Wuhan, China; its water content was 92.5%. The preliminary analyses of biomass used in multi-insect biorefinery are presented in Table 1.

**Table 1:** Preliminary characteristic of corn stover and carrot in dry mass

Parameters	Corn stover	Waste carrot
Crude fat (%)	4.61 ± 0.05	ND
Crude protein (%)	8.49 ± 0.07	ND
Cellulose (%)	32.49 ± 1.62	4.32 ± 1.28
Hemicellulose (%)	30.5 ± 0.80	4.29 ± 0.59
Lignin (%)	9.76 ± 0.10	10.26 ± 1.09
Water content (%)	0	92.50 ± 0.16

*ND* not determined

### Experimental strategy and process

#### First stage of multi-insect biorefinery

1700 YMW larvae (38.72 g) was introduced into 200 g dry mass CS feeding/bedding substrate and reared under laboratory conditions—at the temperature of 28 ± 2 °C and the relative humidity at 70% ± 5%. The experiment was done in triplicates. The CS substrate was provided twice (100 g per addition) to the YMW larvae during the development. Waste carrots (13 g wet weight) were sliced on the top of bedding substrate every 2 days for a total weight of 229.25 g (29.25 g dry mass) over the 63 days of feeding *T. molitor*. YMW larvae were separated from the residues, washed with distilled water, and inactivated at 105 °C for 5 min, and then dried at 60 °C for 2 days. Dried larval biomass was used for grease extraction and biodiesel production.

#### Second stage of multi-insect biorefinery

Residues from the first stage containing YMW frass and remaining CS were used for BSF larval development. During this process, 400 BSF larvae were introduced into 750 g residues (150 g dry mass) in the greenhouse at 27 °C and 70% relative humidity. The experiment was done in triplicates. The investigation was terminated when 50% of BSF larvae had developed into prepupae. Mature BSF larvae were separated from the residues and dried as described for the YMW larvae. The BSF larval biomass was processed for biodiesel production. The residues after the first-stage treatment with *T. molitor* larvae and the second-stage treatment with *H. illucens* larvae were sampled in triplicates for further analysis.

#### Control experiment using BSF larvae only

To make a comparison, evaluating the enhanced efficiency of multi-insect biorefinery, 200 g dried CS (800 g wet substrate) was used for conversion by only BSF, without YMW pretreatment. Proportionally, 400 BSF larvae (same age) were inoculated into the prepared matrix and then incubated in a greenhouse under the same conditions as described above. The experiment was done in triplicates. The experiment was ended when about 50% of the larvae turned into prepupae. Mature BSF larvae were separated from the residues and dried for further analysis.

### Crude grease extraction

To determine the fat content and enhance the fat yield of the larvae in each sample from the biorefinery process, fat removal was achieved employing a previously described method [27]. A classic Soxhlet system with the extraction of cellulose cartridges was utilized. In brief, samples were subjected into 200 mL petroleum ether in a Soxhlet extractor and extracted twice for 8 h. Then crude grease was obtained by combining the leaching solution and evaporating petroleum ether with the rotary evaporator. The crude grease from each sample was calculated according to the weight and used for the further process.

### Biodiesel production from larval biomass from multi-insect biorefinery process

The extracted grease contained various kinds of impurities. Therefore, it was necessary to be purified by adding 0.5% H_2_SO_4_. A two-step method: acid-catalyzed esterification of free fatty acids (to decrease the acidity of the crude grease), and alkaline-catalyzed transesterification was chosen for biodiesel production [26, 27]. A two-step process produced biodiesel because of the high free fatty acid content in grease from multi-insect biorefinery [35, 36]. The reaction was conducted in a reactor equipped with a reflux condenser, a thermometer, mechanically stirring and sampling outlet. Free fatty acids were transformed into biodiesel, which reduced the acidity of crude oils, and the free fatty acids decreased to less than 0.1% after esterification. The mixture was poured into a funnel for separating. The upper layer was transferred to the reactor for alkali catalyzed transesterification as previously described. In brief, to a 6:1 ratio of fat and methanol, 0.8% (w/w) NaOH was added, and the mixture was placed in 65 °C water bath for 30 min, stirring with a magnetic stirrer. After the reaction, the mixture was separated by gravity in a hopper, and the upper layer was purified from the lower, at 80 °C, to remove the residual methanol [23].

### Fatty acid composition and fuel properties analysis of biodiesel

To investigate the fatty acids’ composition of the biodiesels produced from multi-insect biorefinery system, i.e., YMW biodiesel and BSFL biodiesel, gas chromatography–mass spectrometric (GC–MS) analyzer (Agilent Technologies, USA) equipped with an HP-5MS column (length: 50 m; diameter: 0.25 mm; film thickness: 0.25 μm) was applied. The oven temperature program used was as follows: isothermal heating at 50 °C for 1 min, then increased to 250 °C at a rate of 5 °C/min, and held there for 10 min. Electron ionization mode (ionization energy of 70 eV) was used for GC–MS detection. The injector and MS transfer temperatures were both set at 250 °C. The scanning mass range selected was 35–550 *m/z*. Each sample was tested in triplicates.

Fuel properties of the obtained fatty acid methyl esters (FAMEs) were determined according to the ASTM standard methods proposed by Wuhan Baier RunJia lubricating oil Co. Ltd., China, and then compared with European biodiesel standard EN 14214 specifications for performance assessment. Biodiesel obtained from various feedstocks could be used in diesel engine only on satisfying the condition that it complies with these standards. Some critical fuel features such as kinetic viscosity, density, cetane number, flash point, cloud point, and acid number were measured to investigate whether the larval grease resulting from insect biorefinery is suitable to produce biodiesel or not. In brief, kinetic viscosity is the average of two acceptable determined values which are the products of the measured flow time and the calibration constant of the viscometer (ASTM D-445); density was determined by hydrometer method (ASTM D-1298); cetane number was determined by four-variable equation (ASTM D-4737); and flash point was determined by Pensky-Martens closed cup tester (ASTM D-93). The cloud point is the temperature at which a cloud is first observed at the bottom of the test jar (ASTM D-2500), and the acid value of crude fat was determined by titration with potassium hydroxide (ASTM D-664).

### Scanning electron microscope (SEM) detection

The surface morphologies of fibers in native CS and residues after first-stage treatment with YMW and the remaining wastes after the second-stage conversion with BSF were prepared for SEM observation to compare the structural differences to verify the possibility and efficiency of degradability in multi-insect-based biorefinery system. Micrographs of characteristic structural changes in the surface of CS were taken by SEM (Hitachi s-4800, scanning electron microscopy, Japan) under an accelerating voltage of 3.0 kV, at two magnifications of 200× and 700×, to display the structural variations. The change in the surface morphology of the fibers was detected using the high-vacuum secondary electron detection technique.

### Chemical and statistical analyses

The contents of cellulose, hemicellulose, and lignin in original and treated CS were analyzed using a semiautomatic fiber analyzer (A200i, ANKOM Technology Co., Ltd, USA) as previously described [37]. The fiber content data recorded in the original CS and waste carrots are presented in Table 1. This measurement comprised three procedures: neutral detergent fiber (NDF), acid detergent fiber (ADF), and acid detergent lignin (ADL) conducted successively twice. The lipid values of CS and residues after the digestion with multi-insect-based biorefinery were measured by weighing both the original and residues samples before and after extraction, with the difference in the sample weights taken as lipid values [38]. Determination of protein contents from CS and residues after insects’ digestion was conducted by automatic Kjeldhal apparatus (Kjeltech 8400) [27].

Bioconversion, feed-conversion ratio (FCR), and waste mass reduction (WMR) on dry mass base were calculated as per the formulas: bioconversion = total larval biomass/feed added × 100%; FCR = weight of ingested food/weight gained; and WMR = *W*_1_ − *W*_2_/*W*_1_ × 100%, where *W*_1_ = weight of original materials and *W*_2_ = weight of residue.

SPSS 16.0 (SPSS Inc., Chicago, IL, USA) was used for statistical analyses. The variation and regression analysis were carried out using Excel software. All results were expressed as mean ± SE. Experimental graphs and curves were drawn by the Origin software version 9.0.

## Results

### First-stage biorefinery: biomass yield, waste reduction, and feed conversion

Utilization of CS by YMW led to fresh insect biomass of 53.57 g and dry larval mass of 15.78 g (Table 2). Therefore, dry larval mass and water content were 29.43 and 70.57%, respectively (Table 3). The larval mass gains were 14.85 and 4.37 g on wet and dry mass basis, respectively. Moreover, 200 g dry CS and 29.25 g dry mass of waste carrot were reduced to 182.09 g by the transformation with YMW. It was noted that the material reduction rate reached 20.57% (Table 2). FCR of the YMW on CS was 10.79 (Table 2).

**Table 2:** Insect biomass yield, waste mass reduction, and feed-conversion ratio of multi-insect biorefinery system operated with YMW and BSF and comparison to single-species conversion

	Initial larval mass (g)^Ww^	Total biomass of final larvae (g)^Ww^	Total biomass of final larvae (g)^Dw^	Average larval growth rate (g/day)^ww^	Larval mass gain (g)^Dw^	Final larvae water content (%)	Dry mass reduction rate (%)	FCR
First stage (YMW)^a^	38.72 ± 0.01	53.57 ± 1.39	15.78 ± 1.03	0.00014 ± 0.97	4.37 ± 0.59	70.57 ± 1.13	20.57 ± 1.77	10.79 ± 1.24
Second stage (BSF)^b^	0.74 ± 0.03	19.64 ± 2.51	4.28 ± 0.41	0.0018 ± 0.51	4.12 ± 0.41	78.21 ± 1.60	38.72 ± 1.01	17.16 ± 0.46
Control (only BSF)^c^	0.88 ± 0.01	12.40 ± 0.02	2.88 ± 0.01	0.0014 ± 0.12	2.67 ± 0.05	76.81 ± 2.28	39.89 ± 0.02	28.59 ± 3.32

*Ww* wet weight, *Dw* dry weight

^a^First stage of multi-insect biorefinery of corn stover using YMW

^b^Second stage of multi-insect biorefinery of corn stover using BSF

^c^Only BSF applied for corn stover transformation as a control for comparison

**Table 3:** Comparative data of development time and dry mass gain from previous reported experiments

Feed source	Species	Development time (day)	Dry mass (%) of live insect larvae	Water content (%) of live larvae	References
Corn stover	*Tenebrio molitor*	63	29.43	70.57	This study
Corn stover	*Hermetia illucens*	26	21.79	78.21	This study
Rice straw	*Hermetia illucens*	38.20–54.20	NA	NA	[17]
Artificial feed	*Hermetia illucens*	21–37	32.9–35.6	64.4–67.8	[39]
Artificial feed	*Tenebrio molitor*	83–227	30.2–41.5	69.8–58.5	[39]
Hen feed	*Hermetia illucens*	15	NA	NA	[40]
Meat meal	*Hermetia illucens*	33	NA	NA	[40]
Dairy manure	*Hermetia illucens*	31	NA	NA	[26]
Chicken feed	*Hermetia illucens*	16–42	33–40	60–67	[41]
Dairy manure	*Hermetia illucens*	~ 120	NA	NA	[42]
Dairy manure	*Hermetia illucens*	26–30	NA	NA	[43]
Commercial diet	*Hermetia illucens*	NA	27	73	[24]
Chicken manure	*Hermetia illucens*	NA	17	83	[44]

*NA* not available

### Second-stage biorefinery: biomass yield, waste reduction, and feed conversion

It was recorded that BSF larvae wet mass and dry mass were 19.64, 4.28 g, respectively. Therefore, it can be concluded that 78.21% was water content of BSF larvae (Table 2). The wet larval biomass gained 18.90 g; whereas the dry larval mass gain was 4.12 g. Moreover, the 182.09 g dry residues from the first stage reduced to 111.59 g with the digestion by BSFL; the material reduction rate reached 38.72% (Table 2), and the feed-conversion efficiency was 17.16.

### Multi-insect biorefinery overall efficiency evaluation

The overall CS and carrot dry mass reduction in the multi-insect biorefinery was 51.32%, and bioconversion and FCR of the refinery were 3.70% and 13.86, respectively (Table 4). The waste reduction, bioconversion, and FCR are in concord with published data (Table 4), demonstrating that multi-insect biorefinery system is a good indicator of the aspect of CS utilization.

**Table 4:** Comparison of different parameters about material reduction rate, biomass-conversion, and feed-conversion ratios in different studies

Feed source	Species	Waste reduction (%)	Bioconversion (%)	FCR	References
Corn stover	*Hermetia illucens*	39.89^Dw^	1.39	28.59	This study
Corn stover	*Tenebrio molitor* and *Hermetia illucens*	51.32^Dw^	3.70	13.86	This study
Rice straw	*Hermetia illucens*	9.58–31.53^Dw^	NA	NA	[17]
Artificial feed	*Tenebrio molitor*	NA	NA	3.80–19.10	[39]
Human feces	*Hermetia illucens*	25.00–55.00^Ww^	2.10–22.30	2.00–15.60	[45]
Swine manure	*Musca domestica*	67.20^Ww^	NA	NA	[46]
Municipal organic waste	*Hermetia illucens*	68.00^Dw^	12.00	14.50	[47]
Swine manure	*Hermetia illucens*	39.00^Dw^	4.00	9.60	[47]
Chicken manure	*Hermetia illucens*	≈ 50.00^Ww^	3.70	13.40	[48]
Chicken and cow manure	*Musca domestica*	25.00^Ww^	NA	10.00	[49]

*Ww* wet weight, *Dw* dry weight, *NA* not available

### Efficiency comparison of multi-insect biorefinery with single-species conversion

As shown in Table 2, BSF larvae in the second stage of multi-insect biorefinery system had a better growth performance compared with the only BSF applied without the predegradation by YMW larve. Although initial larval average weight of the control was a little higher than that of the second stage’s BSF (0.0022 and 0.0019 g, respectively), a higher final larval average weight (0.049 g), average larval growth rate (0.0018 g/day), and bioconversion rate (2.26%) were obtained when multi-insect biorefinery was applied. This indicated that CS predigested by YMW was more suitable for BSF larvae utilization and could reach a higher insect biomass production rate.

It can also be seen that the dry mass reduction rates from both were comparable, which increased marginally when the raw CS was digested only by the BSF in the control group (Table 2), but it was far below the total waste reduction rate of multi-insect biorefinery established in this study (39.89 and 51.32%, respectively) (Table 4). Moreover, much higher FCR was obtained for the control group compared with the new biorefinery system, which means more raw materials will be required to produce equal insect biomass. Hence, it could be concluded that multi-insect biorefinery became more efficient when YMW and BSF were applied successively.

### Fluctuation in reducing sugar, lipid, and protein during multi-insect biorefinery

**Fig. 1:**
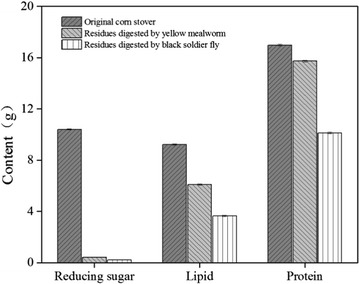
Dynamic changes of reducing sugar, lipid, and protein during multi-insect corn stover degradation process

The utilization efficiencies during the multi-insect biorefinery of different components such as reducing sugar, fat, and protein in the samples were analyzed. Figure 1 demonstrated that reducing sugar declined primarily after the first-stage biorefinery, from 10.40 to 0.42 g. However, the lipid and protein contents only varied slightly, from 9.22 to 6.1 g and 16.98 to 15.73 g, respectively. The reducing sugar decreased slightly in second-stage biorefinery, from 0.42 to 0.22 g, whereas lipid reduced from 6.1 to 3.64 g and protein from 15.73 to 10.12 g. The aggregate degradation rates of reducing sugar, lipid, and protein in the whole multi-insect biorefinery process were 97.88, 60.52, and 40.40%, respectively.

### Variation in lignocelluloses throughout multi-insect biorefinery

**Fig. 2:**
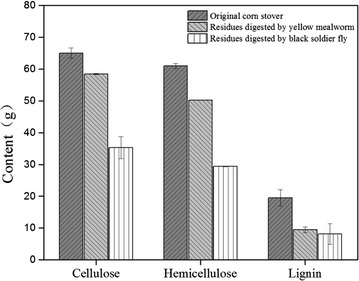
Reduction rates of cellulose, hemicellulose, and lignin in corn stover during multi-insect biorefinery process

The cellulose, hemicellulose, and lignin content in the CS were 64.98, 61.00, and 19.52 g. The cellulose declined from 64.98 to 58.41 g after YMW conversion, and after digestion, with BSFL only 35.29 g cellulose remained. Similarly, hemicellulose decreased by the action of YMW from 61.00 to 50.22 g, and after the second-stage BSFL digestion, only 29.37 g remained (Fig. 2). The lignin utilization was higher by the first-stage YMW utilization (reduced from 19.52 to 9.49 g) as compared to the second-stage BSFL digestion (reduced from 9.49 to 8.13 g) (Fig. 2).

### Morphological characterization of corn stover fibers by SEM

**Fig. 3:**
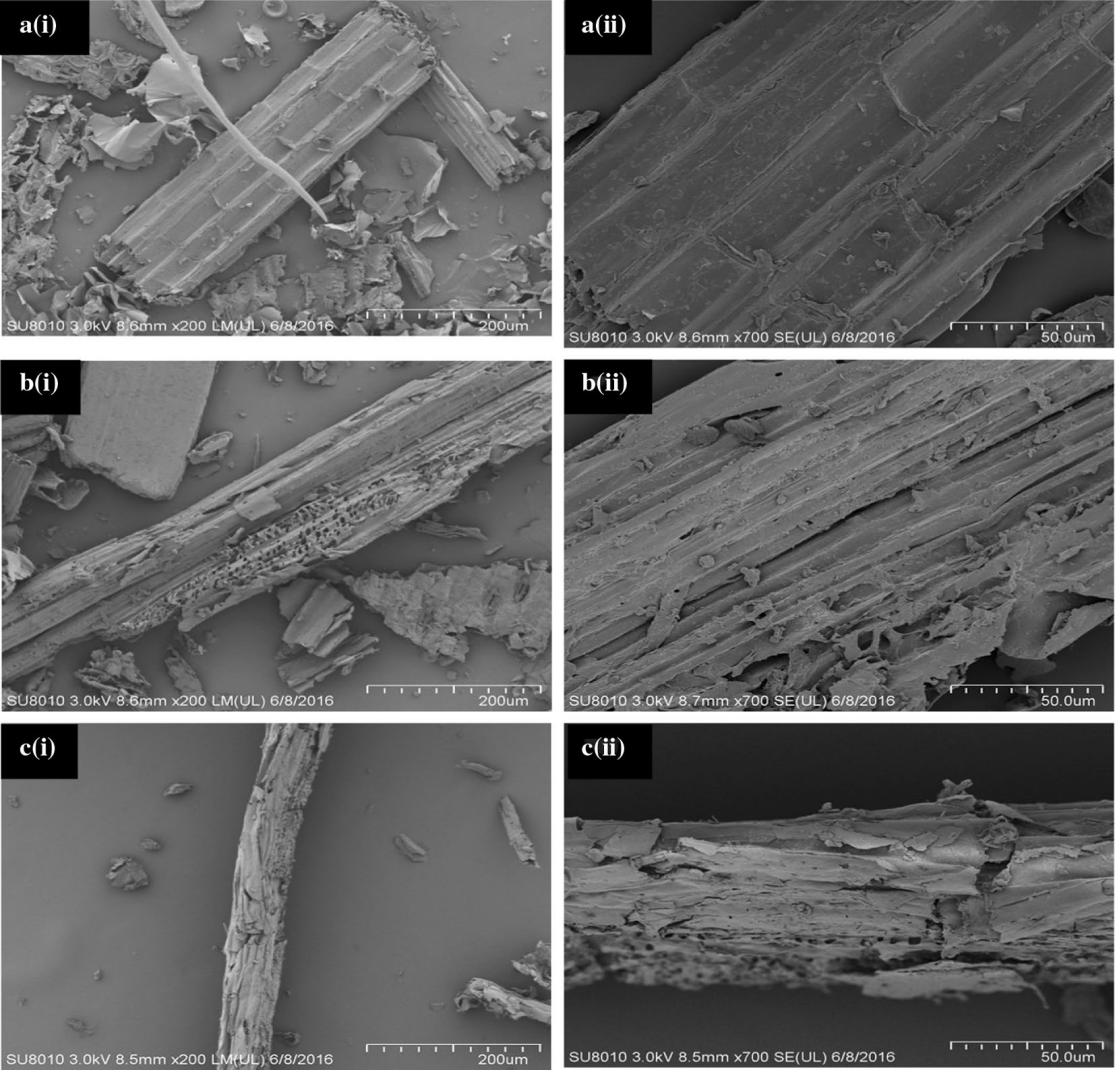
Scan electron micrographs of fiber **a** original corn stover, **b** residues digested by yellow mealworm (first-stage biorefinery), and **c** residues digested by black soldier fly (second-stage biorefinery), at (i) ×200 magnification and (ii) ×700 magnification

Before treatment, CS surface structure was compact, plain, smooth, and well organized (Fig. 3a). First-stage YMW utilization of CS resulted in the substantial destruction of the CS surface that extended into the vascular bundles and adjoining cells, i.e., parenchyma cells (Fig. 3b). The surface of the CS become more rough, hollow, cracked, and porous compared with the original CS. Likely YMW nibbled the stem cell wall causing the change to the small granules during digestion. The subsequent BSFL degradation of the remaining residues is documented in Fig. 3c, and it demonstrated that the structural matrix of CS was distorted entirely and cracked; hence, it can be assumed that BSF is capable of digesting fibers from crop residues.

### Biodiesel production from the multi-insect biorefinery system

**Fig. 4:**
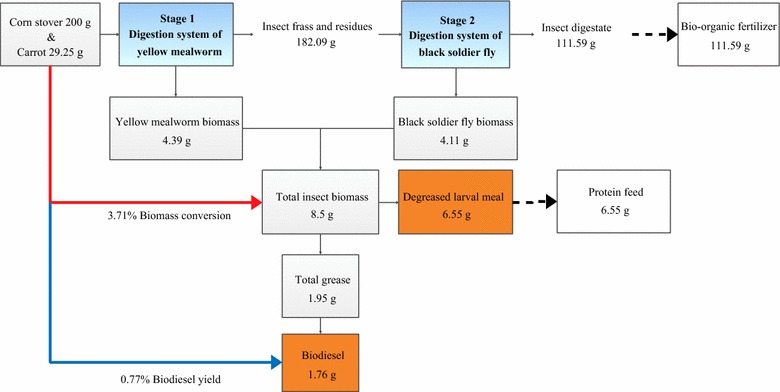
Material flow in multi-insect bioconversion process of lignocelluloses: brown boxes with black text indicate the actual product obtained in the current article; dashed lines indicate pathways for value-added byproducts

The material flow in multi-insect waste utilization biorefinery process is described in Fig. 4. The consumption rate of CS was 51.32% after utilization of the two species, YMW and BSF (Table 4). This consecutive processing system of waste management and the use of resources by 1700 YMW larvae and 400 BSF larvae produced 1.95 g grease and 1.76 g biodiesel (Fig. 4).

This larval grease obtained from multi-insect biorefinery was found to be suitable for serving as raw material for biodiesel production. The chemical compositions of fatty acid methyl esters in YMW biodiesel and BSFL biodiesel from multi-insect corn stover biorefinery were analyzed by GC–MS mentioned above, and the results are shown in Table 5. The numbers of carbons of fatty acids present in the YMW biodiesel and BSFL biodiesel ranged from 8 to 20, and 10 to 19, respectively, which were similar to that of fossil diesel. As shown in Table 5, eight different fatty acids were detected in YMW biodiesel, including lauric acid (47.47%), myristic acid (27.62%), and linoleic acid (11.54%) as the main components. About 10 different fatty acid methyl esters were detected in BSFL biodiesel, among which lauric acid (57.28%) was the predominant ingredient, followed by hexadecenoic acid (12.15%), and linoleic acid (6.7%); some ordinary fatty acids like myristic acid (6.58%), decanoic acid (6.15%), and palmitic acid (5.65%) also were found in BSFL biodiesel. Moreover, properties of YMW and BSFL grease-derived biodiesels were comparable to those of European biodiesel standard (EN14214), biofuels derived from waste cooking oils and feed crops (Table 6).

**Table 5:** Relative contents of fatty acids’ compositions of biodiesels from different feedstocks

Fatty acids	YMW biodiesel (this study)	BSFL biodiesel (this study)	YMW biodiesel [23]	BSFL biodiesel [26]	Rapeseed biodiesel [50]
Octanoic acid (8:0)	0.30 ± 0.02	ND	NA	NA	NA
Decanoic acid (10:0)	0.68 ± 0.06	6.15 ± 0.18	1.20	3.10	NA
Lauric acid (12:0)	47.47 ± 3.83	57.28 ± 2.74	1.30	35.60	NA
Myristic acid (14:0)	27.62 ± 0.84	6.58 ± 0.39	8.10	7.60	NA
Pentadecanoic acid (15:0)	ND	0.19 ± 0.04	1.50	–	NA
Palmitic acid (16:0)	4.16 ± 0.31	5.65 ± 0.95	17.60	14.80	3.49
Hexadecenoic acid (16:1)	ND	12.15 ± 0.15	9.30	3.80	NA
Heptadecanoic acid (17:0)	ND	ND	1.70	1.00	NA
Stearic acid (18:0)	5.39 ± 0.85	ND	11.40	3.60	0.85
Oleic acid (18:1)	ND	ND	1.60	23.60	64.40
Linoleic acid (18:2)	11.54 ± 3.70	6.70 ± 0.29	16.30	2.10	22.30
Linolenic acid (18:3)	ND	0.16 ± 0.23	19.70		8.23
Nonadecanoic acid (19:0)	ND	ND	2.60	1.40	NA
Nonadecenoic acid (19:1)	ND	1.09 ± 0.05	NA	NA	NA
Eicosenoic acid (20:1)	2.82 ± 0.32	ND	NA	NA	NA

*NA* not available, *ND* not determined

**Table 6:** Comparison of biodiesel properties derived from YMW larvae fed on corn stover and BSF larvae fed with YMW frass with European biodiesel standard (EN14214), and biodiesels derived from waste cooking oils and feed crops

Properties	YMW biodiesel	BSF biodiesel	EN14214	Mixed waste cooking oil biodiesel [51, 52]	Waste sunflower oil [52]	Neem oil biodiesel [53]	Cotton seed oil biodiesel [54]	Soybean biodiesel [55]	Corn biodiesel [55]
Density (kg/m^3^)	876	872.8	860–900	860	888	871	864	885	880
Viscosity at 40 °C (mm^2^/s)	4.51	3.284	3.5–5.0	4	4.42	4.63	4.14	4.08	3.4
Sulfur content (wt%)	ND	ND	0.02 (max.)	NA	NA	NA	NA	NA	NA
Ester content (%)	97.2	96.9	97	98.2	99.5	NA	NA	NA	NA
Water content (mg/kg)	260	180	500 (max.)	NA	NA	NA	NA	NA	NA
Flash point (°C)	156	122	120 (min.)	155	NA	NA	NA	NA	NA
Solidifying point (C)	− 2	− 8	NA	NA	NA	NA	NA	NA	NA
Cetane index	52	50	51 (min.)	56	51.4	53.5	52	52	58
Acid number (mg KOH/g)	0.27	0.2	0.5 (max.)	NA	NA	NA	NA	0.15	NA
Distillation temperature (°C)	362	355	NA	NA	NA	NA	NA	NA	NA

*NA* not available, *ND* not determined

## Discussion

The novel concept of insect-based biorefinery utilizing agricultural waste as a raw material promises an alternative to fossil resources to produce biodiesel and protein, thereby mitigating climate change and improving energy, feed, and food security. The present study highlighted the possibility of a multi-insect biorefinery for production of biodiesel and protein, converting agricultural residues into insect biomass. The biorefinery studied includes initial CS degradation by YMW, followed by a second stage that engaged BSF, to utilize the wastes produced during the first stage. Sustainability is becoming more and more important in the world. An alternative to protein and oil must be found to replace traditional, unsustainable ingredients. One possible alternative to conventional proteins and oils is insects (YMW and BSF) [56], the protein used for compound feed formulation in livestock and fats, which can be used for biodiesel production.

Previous studies have demonstrated that YMW can convert wheat bran, corn flour, fodder, and crop residues, likely because it can digest the crude fiber present in the plant’s residues [57, 58], and the lignocellulose-rich CS residues were used to run this insect-based biorefinery. The dry larval biomass and water content of YMW larvae were in agreement with prior investigations (Table 3). It has been previously noted that the defective carrots from vegetable market waste were utilized to meet the moisture consumption for *T. molitor* larval biomass development by adding slices of the fruits [59], and the similar practice, by adding slices of defective carrots wastes, was adopted in this study. It can be observed that defective waste carrots used have 92.5% water content, so it can be adequate to satisfy the requirement of water for the development of YMW larvae. YMW used CS less efficiently (FCR = 10.57) compared to the artificial feed (Table 4), but in agreement with that when less-nutrient and high-water contents are offered for development, the FCR ranges from 3.80 to 19.1 (Table 4). Diet composition is the primary variable for determining feed-conversion efficiency for given insect species. Therefore, the presence of cellulose, hemicellulose, and lignin in waste carrots (Table 1) (4.32, 4.29, and 10.26%, respectively) may have certain effects on the larval biomass development. Li et al. [58] reported that the crude fiber content in insect feed affects the life-history traits and biomass development.

The second-stage BSF biorefinery was first investigated for biomass production and derivation of liquid bioenergy based on the first-phase residues. Previously, many studies on utilizing crop straws, vegetable waste, and other organic wastes were performed by only one kind of insect species, either by YMW larvae [20, 23, 59] or BSF larvae [17–19, 26–28, 60, 61]. Therefore, in this investigation for improved management of natural resources, BSFL was further employed to convert the first-stage insect frass residues. The water content of the BSFL in the previously reported studies was 60–67% [41] when developed on commercial chicken feed, and 73.2% [24] on a commercial diet (Table 3). An interesting finding was observed when chicken manure with relatively high water content was used: it resulted in the higher water content and dry mass of 83% and 17%, respectively [44]. Therefore, the higher water content of larvae may be due to the water supplemented when the percentage of moisture of substrate became < 70%, (or < 60% at 3 days before the termination of the experiment). The FCR was in agreement with prior investigations on BSF fluctuating from 2.0 to 19.10 (Table 4). The efficiencies of the insect, such as biomass development, bioconversion, and feed-conversion efficiencies, depend on the diet used for their development [39, 62]. It was also noted that BSF larvae in the second stage of multi-insect biorefinery system had a better growth performance compared with the only BSF being applied without the predegradation by YMW larvae.

The reducing sugars, lipids, and protein contained in the native CS were 9.22, 10.4, and 16.98 g, respectively (Fig. 1). The relative percentages were 5.2, 4.61, and 8.49%, respectively, in dry mass; these recorded values were identical with the previous analysis of reducing sugar [63], lipids, and protein [64] for CS. The degradations of reducing sugars, lipids, and protein from the waste for insect biomass development were previously reported from rice straw and restaurant waste by BSFL [27, 61], and the reduction rate of the nutrient depends upon the waste material offered for insect development [25]. In summary, these components decreased during the successive conversion of the multi-insect system.

The cellulose, hemicellulose, and lignin contents in the CS were 64.98, 61.00, and 19.52 g, respectively. The relative percentages of three principal components were 32.48, 30.50, and 9.76%, respectively, which are similar to the previous estimation of fiber content in CS [65, 66]. The total multi-insect degradation rates of cellulose, hemicellulose, and lignin were 45.69, 51.58, and 58.35%, respectively. Overall, the lignin was mainly digested in the first stage of insect degradation, whereas cellulose and hemicellulose were primarily utilized in the second stage by BSF. The higher lignin and lower cellulose deteriorations in the first stage may be due to the lignin-derived phenolic compound generated during lignocellulosic biomass hydrolysis that inhibits cellulose conversion [67–70]. The utilization and reduction of lignocellulose by the insects reflect the gut microbiota present in the insects [18, 58, 68].

The YMW utilizations of cellulose, hemicellulose, and lignin in this study were based on the recent findings of *T. molitor* digestion of dietary fibers [58], and natural polymer utilization by mealworms [67, 71]. The utilizations of cellulose, hemicellulose, and lignin by BSF were previously noted in dairy manure [19, 26], corncob [29], rice straw [27], and soybean curd residues [18], but the corresponding utilization efficiency was relatively small. Successive multi-insect biorefinery technology employing YMW and BSF enhanced the overall CS conversion rate, which resulted in the higher waste-to-energy efficiency.

SEM was used to record the destruction of the cell wall structure of CS during the process of multi-insect biorefinery. The degraded product is used for the productions of biofuel and animal grade feed. SEM is a valuable tool that has been extensively employed for morphological inspection that describes the surface profile of an object. In order to obtain a good description of an object, the object should be metallic. Therefore, to analyze the surface of CS, they should be coated with metallic strips and metal, such as palladium gold. Structural changes in the surface morphology of corn cell wall cellulose during the enzymatic action were formerly recorded by SEM [11].

The crude grease was extracted from the larvae by Soxhlet extraction [23, 26]. The conversion rate of free fatty acids in crude grease into biodiesel reached about 90% in the present investigation. However, the mean total yield of biodiesel was approximately 1.99% of the waste biomass, which is marginally less, but still comparable, to the productivities of biodiesel from crude grease and waste biomass of 91 and 2.4%, respectively [28]. This may be due to the higher fiber content of CS that interferes with larval development versus that of the restaurant waste investigated in the former study. SEM micrographs documented the damage to the crop cell walls by insect utilization in this study. These changes may be due directly to the insect, or to their microbiome, which has been previously shown to be able to digest the lignocellulose biomass by cellulolytic enzyme [72].

The fatty acids’ compositions of five different biodiesel obtained from various feedstock are compared with YMW and BSF larval fat-derived biodiesel in Table 5. Compared with YMW larvae fat-based biodiesel fed on decayed vegetables [1], YMW biodiesel from the present study possessed unique octanoic acid and eicosenoic acid compositions with relative contents of 0.30 and 2.82%, respectively, and the absence of pentadecanoic acid, hexadecenoic acid, oleic acid, linolenic acid, and nonadecanoic acid. Moreover, the composition and content of BSF larvae fat-based biodiesel fed on digested residues in this work were similar to those in Li’s study [2], except for a few changes in those of pentadecanoic acid and oleic acid. Biodiesel produced from plant seed-like rapeseed had an uncomplicated fatty acid composition mainly composed of 18 C unsaturated fatty acid [3]. The relative contents of saturated fatty acids and unsaturated fatty acids in YMW biodiesel were 85.62 and 14.34%, while in BSFL biodiesel, these were 77.05 and 20.1%, respectively.

It was noted that the biodiesels derived from the YMW larvae that fed on CS and the BSF larvae that fed with frass from the first stage of insect production have similar performance properties. The properties of the biodiesel, from YMW and BSFL larvae, met with the fuel specifications of EN14214, including density (876.3 and 872.8 kg/m^3^), ester content (97.2 and 96.9%), water content (260 and 180 mg/kg), cetane index (52 and 50), acid number (0.27 and 0.20 mg KOH/g), flash point (156 and 122 °C), etc. Therefore, the biodiesel characteristics from both stages of biorefinery were similar to those described by Zheng et al. [23].

The viscosity at 40 °C and a flash point of YMW grease-based biodiesel were slightly higher than BSF biodiesel, and within an acceptable range as specified by standard EN14214. Higher viscosities were noted in a previous investigation for both YMW and BSF larvae, so biodiesel blending with petrodiesel is recommended for actual use [23]. In this investigation, the viscosity was reasonable for synthetic diesel and matched with the European standards (Table 6). The biodiesel flash point was greater than diesel; methanol content is one of the important factors for higher biodiesel flash point [23, 27, 28]. The acid numbers of YMW- and BSF-derived biodiesel recorded were in accordance with the EN14214 standards, but in prior studies, the acid number was higher in the biodiesel derived from similar insect species [23].

## Conclusion

This investigation demonstrated the process of biodiesel production from lignocellulose by a multi-insect biorefinery. The multi-insect utilization of lignocellulose comprises a first-stage YMW larvae-developed larval biomass with utilization of CS, followed by the second stage of BSF larvae on the frass from the first stage to utilize lignocelluloses resources more efficiently. The novel idea was successfully tested in this study. The YMW and BSF larvae’s biomass was further used to produce biodiesel for liquid energy needs and defatted larval meal for animal feed protein supplement. It is worth stating that the process in this study reached a satisfactory conversion rate and product yield without the requirement of any chemical reagents or material pretreatments which threaten the environment. Therefore, the process was considered as a green environmentally friendly technology.

Biodiesel production from insects developed on organic waste has great potential to satisfy the increasing demands for liquid fuels, particularly in developing continents (i.e., Asia, Africa, and Middle-east). It will cut fossil energy consumption, relieve the impact on the environment, and reduce the cost of biodiesel. This investigation employed an innovative environmentally friendly technology by applying two insects consecutively to improve the CS utilization to produce biodiesel, defatted larval meal, and biofertilizer. However, before such insect biorefinery is implemented successfully as a green technology, it is still necessary to test the biodiesel derived from the insect’s biomass and analyze its performance and emissions on the diesel engine.

## Abbreviations

b/d: barrels per day
Btu: British thermal unit
CS: corn stover
YMW: yellow meal worm
BSF: black soldier fly
BSFL: black soldier fly larave
HZAU: Huazhong Agricultural University
SEM: scanning electron microscope
FCR: feed-conversion ratio
WMR: waste mass reduction
Ww: wet weight
Dw: dry weight
NA: not available
ND: not determined
